# Embryonic development of circadian clocks in the mammalian suprachiasmatic nuclei

**DOI:** 10.3389/fnana.2014.00143

**Published:** 2014-12-01

**Authors:** Dominic Landgraf, Christiane E. Koch, Henrik Oster

**Affiliations:** ^1^Center of Circadian Biology and Department of Psychiatry, University of California, San Diego, and Veterans Affairs San Diego Healthcare SystemSan Diego, CA, USA; ^2^Chronophysiology Group, Medical Department I, University of LübeckLübeck, Germany

**Keywords:** suprachiasmatic nucleus, circadian clocks, embryonic and fetal development, entrainment, clock genes

## Abstract

In most species, self-sustained molecular clocks regulate 24-h rhythms of behavior and physiology. In mammals, a circadian pacemaker residing in the hypothalamic suprachiasmatic nucleus (SCN) receives photic signals from the retina and synchronizes subordinate clocks in non-SCN tissues. The emergence of circadian rhythmicity during development has been extensively studied for many years. In mice, neuronal development in the presumptive SCN region of the embryonic hypothalamus occurs on days 12–15 of gestation. Intra-SCN circuits differentiate during the following days and retinal projections reach the SCN, and thus mediate photic entrainment, only after birth. In contrast the genetic components of the clock gene machinery are expressed much earlier and during midgestation SCN explants and isolated neurons are capable of generating molecular oscillations in culture. *In vivo* metabolic rhythms in the SCN, however, are observed not earlier than the 19th day of rat gestation, and rhythmic expression of clock genes is hardly detectable until after birth. Together these data indicate that cellular coupling and, thus, tissue-wide synchronization of single-cell rhythms, may only develop very late during embryogenesis. In this mini-review we describe the developmental origin of the SCN structure and summarize our current knowledge about the functional initiation and entrainment of the circadian pacemaker during embryonic development.

## Introduction

Endogenous circadian clocks facilitate the adaptation of behavior and physiology to the 24-h rhythm of day and night. In mammals, the circadian timing is organized by pacemaker cells in the hypothalamic suprachiasmatic nuclei (SCN). This SCN master clock is reset by photic time cues, or *Zeitgebers*, perceived through the retina and transmitted via the retino-hypothalamic tract (RHT). At the molecular level, the cellular clocks in the SCN and other tissues are built from self-sustained interlocked transcriptional-translational feedback loops of clock genes/proteins characterized by rhythmic transcription patterns. While clock function and rhythm generation have been extensively studied in adults, there is still no agreement on how circadian rhythms emerge during embryonic development.

## Anatomical development of the SCN

**Figure 1:**
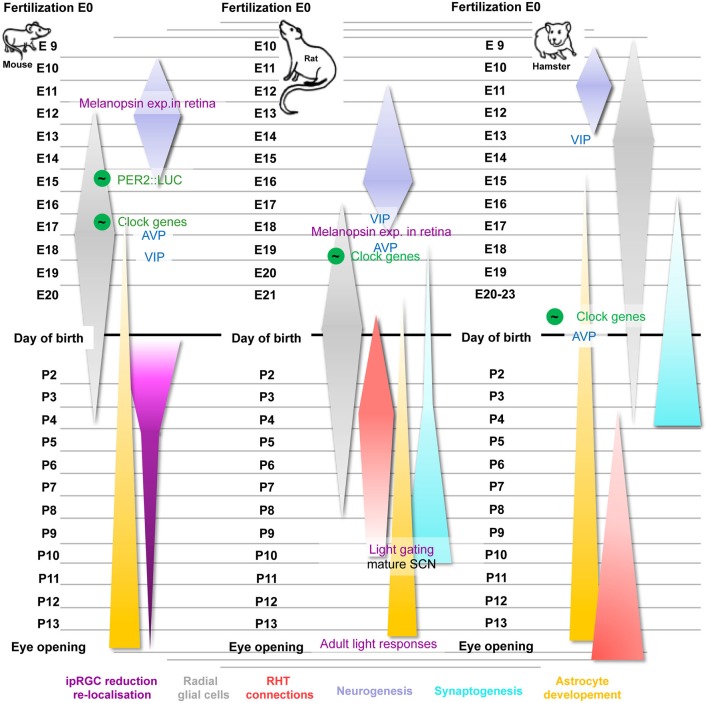
**Time course and critical steps of SCN development in mice, rats and hamsters**. Important steps of circadian development use green color for all species (the green-circled wave symbol indicates rhythmicity), typical SCN-related proteins are in blue and events related to the RHT are shown in purple. Furthermore, the neurogenesis of each species occurring during the embryonic state is highlighted in purple, the ontogeny of radial glia cells in illustrated in gray and astrocyte development in yellow. Synaptogenesis (cyan) and RHT maturation (red) are shown for rats and hamster only. The reduction and re-localization of ipRGCs (pink) were highlighted during the postnatal development of mice. For details see text.

The ontogeny of the SCN has been extensively described in rodents (Figure 1), while only few data on primate SCN development are available (Weinert, 2005). The rat SCN is derived from the neuroepithelium of the preoptic recess of the third ventricle and becomes discernable as a discrete structure at embryonic day E17 (Altman and Bayer, 1978b). Neurogenesis in the rat mainly occurs between E12 and E18 with a maximum at E16 (Ifft, 1972; Altman and Bayer, 1978a). In mice neurogenesis begins earlier and is restricted to days E10–15, peaking at E12 (Shimada and Nakamura, 1973; Kabrita and Davis, 2008). In the hamster SCN, neurons are born even earlier at E9.5–13 (Davis et al., 1990; Antle et al., 2005). In squirrel monkeys, SCN neurons have been described at late gestational stages and, in humans, the SCN is discernable as a discrete structure around the 18th–30th week of pregnancy (Reppert and Schwartz, 1984; Reppert et al., 1988; Swaab et al., 1990).

The ontogeny of non-neuronal cell types within the SCN also follows temporal programs of formation (Botchkina and Morin, 1995; Antle et al., 2005). In the hamster SCN, radial glial cells start to develop at E8 and appear at high density in the SCN at E13. At post-natal day 0 (P0), the density of these cells in the hamster SCN is drastically reduced and by P5 most of them are replaced by neurons. Hamster SCN astrocytes start to form at E15 and development continues at least until P21 (Botchkina and Morin, 1995). In the rat, astrocytes first appear at E20 and rapidly increase after birth, and radial glia cells are present at E17, which was the earliest stage investigated in this study (Munekawa et al., 2000), and begin to disappear at P0.

## Activation of SCN marker gene expression

The neuropeptides vasopressin (AVP) and vasoactive intestinal polypeptide (VIP) are strongly expressed in the SCN and contribute to its role as circadian pacemaker (Vosko et al., 2007; Kalsbeek et al., 2010). In the hamster SCN, AVP expressing cells first appear on the day of birth (Romero and Silver, 1990). In the mouse, AVP neurons first appear at E12 at the peak of neurogenesis (Okamura et al., 1983). However, AVP mRNA appears later at E17.5 of mouse development and AVP protein is only detectable at postnatal stages (VanDunk et al., 2011). From the 27th week of pregnancy, AVP is detectable in the human embryonic hypothalamus (Swaab et al., 1990). From then on, AVP levels constantly increase until 1–1.5 years after birth.

In the developing hamster SCN, VIP expressing neurons are detectable after completion of neurogenesis at E13–14 and VIP expression increases substantially until P10 (Romero and Silver, 1990; Botchkina and Morin, 1995). VIP mRNA first appears only after neurogenesis at E18.5 in the developing mouse SCN and VIP protein is detectable after birth (VanDunk et al., 2011). In contrast, in the rat SCN, *VIP* mRNA first appears directly after completion of neurogenesis at E18 and increases after birth until stage P20 (Ban et al., 1997). VIP protein in the rat SCN is first expressed no later than E20 (Laemle, 1988). However, earlier embryonic stages have not yet been investigated. The first VIP neurons in the human SCN are observed at 31^th^ week of pregnancy with increasing numbers until the age of 3 years. Spatial re-organization of VIP neurons, however, continues throughout puberty (Swaab et al., 1994).

In mice, a combination of transcription factors shows distinct spatial and temporal patterns during prenatal and postnatal SCN development (VanDunk et al., 2011). The SCN anlage emerges from a specific region of the neuroepithelium expressing *Six homeobox 3* and *6* (*Six3*, *Six6*), *frizzled-5* (*Fzd5*), and transient *retinal homeobox gene* (*Rx*). Throughout embryonic development *Six3*, *Lim homeodomain transcription factor 1* (*Lhx1*) and *RAR-related orphan receptor alpha* (*Rora*) expression remains restricted to the SCN region. Conditional deletion of *Six3* at early stages prevents SCN development completely (VanDunk et al., 2011). In addition, *Six6* is required for the normal development of the optic nerves and the SCN. *Six6*-null mice display abnormal entrainment behavior, due to the lack of functional optic nerves, but also show abnormal circadian rhythms under free-running conditions, indicating the absence of a functional SCN. Indeed, immunohistochemical stainings for VIP and AVP confirmed the absence of a defined SCN structure in *Six6*-null mice (Clark et al., 2013). A selective deletion of SCN-enriched *Lhx1* in the developing anterior hypothalamus, including the developing SCN, leads to reduced SCN-enriched neuropeptides involved in circadian function and causes loss and death of neurons in the developing mouse SCN (Bedont et al., 2014). Furthermore, the loss of *Lhx1* during SCN development leads to reduced cellular synchrony in the SCN and results in disrupted circadian rhythms in adult mice (Bedont et al., 2014; Hatori et al., 2014).

## Development of circadian pacemaker function in the SCN

At which embryonic stage endogenous SCN rhythms emerge, is still a matter of debate. Shortly before birth, glucose utilization in the embryonic SCN of squirrel monkeys shows diurnal rhythms (Reppert and Schwartz, 1983, 1984). Similarly, expression of the neuronal activity marker *c-fos* in the embryonic SCN of sheep at gestational day 135 shows diurnal fluctuations (Constandil et al., 1995). However, the mother animals used for these studies had functional circadian clocks and were housed in rhythmic light-dark (LD) conditions during the entire or at least during large parts of gestation. Thus, maternal or external timing cues driving diurnal rhythms observed in fetuses cannot be excluded and the existence of an endogenous fetal circadian clock cannot be concluded from these studies. Contrary, in rats it was shown that embryonic SCN rhythms emerge even in the absence of maternal clocks and under constant housing conditions. Glucose utilization and also neuronal firing of cultured embryonic rat SCNs at E22 exhibit circadian rhythms *in vitro*, suggesting that these oscillations are evoked by an endogenous clock (Shibata and Moore, 1987, 1988). In addition, the offspring of SCN-lesioned rat mothers displays circadian behavioral rhythms indicating that a functional circadian system develops in the absence of rhythmic maternal signals (Reppert and Schwartz, 1986). In humans, preterm infants of 29–35 weeks of age show circadian temperature rhythms under constant lighting and feeding conditions, which suggests the existence of a functional pacemaker during human gestation (Mirmiran and Kok, 1991).

Additional indirect evidence of functional embryonic SCN clocks comes from transplant studies. Implanting embryonic SCN cells or grafts into the brain of SCN-lesioned adult hamsters restores free-running rhythms soon after transplantation (Silver et al., 1990; Kaufman and Menaker, 1993). Importantly, the outcome was independent of the age of the donor embryo suggesting that the SCN is already fully functional during midgestation.

In contrast, examination of clock gene or protein expression in fetal SCNs yields ambiguous results. In constant darkness, mRNA expression of *Per1*, but not *Per2*, is rhythmic at E17 in mice (Shimomura et al., 2001). At E18, PER1 and PER2 protein levels show circadian rhythms in the mouse embryonic SCN when mothers are kept in constant darkness, albeit with low baseline levels and moderate amplitude (Ansari et al., 2009). In rats kept under LD conditions, *Per2* and *Bmal1* mRNA expression is not rhythmic at E19, but on E21 (Houdek and Sumová, 2014). A different study has shown that *Per1* and *Per2* mRNA is already rhythmic in rat embryos at stage E20 (Ohta et al., 2002, 2003). Interestingly, although *Per2* and *Bmal1* expression is arrhythmic at E19 in the rat SCN, *nuclear receptor subfamily 1, group D, member 1* (*Nr1d1*), *c-fos*, *Avp*, and *Vip* mRNAs already show significant circadian rhythms that are likely driven by maternal signals (Houdek and Sumová, 2014). Rhythmic *Bmal1* and *Per2* expression is seen in fetal SCNs of capuchin monkeys at late stages of gestation (Torres-Farfan et al., 2006). However, since the mother animals used in all these studies had a functional circadian clock and/or were kept in an LD cycle, an influence of rhythmic maternal signals on rhythmic clock gene expression in the embryonic SCN cannot be ruled out. Accordingly, other studies have shown that when pregnant rats are kept in constant darkness, *Per1*, *Per2*, and other clock gene mRNAs and proteins are not rhythmic in the embryonic SCN at E19/20 and rhythms only emerge around P1 (Sládek et al., 2004; Kováciková et al., 2006). However, cultured explants of PERIOD2::LUCIFERASE (PER2::LUC) mice, which carry the firefly luciferase gene in the wild-type circadian clock gene *Per2* as a reporter for circadian rhythms (Yoo et al., 2004), revealed the capability of early embryonic SCNs of stage E15 to express self-sustained circadian rhythms *in vitro*. With advancing age of the embryos, the rhythms became gradually stronger (Wreschnig et al., 2014). Whether the observed rhythms reflect circadian oscillations that were already present *in vivo* or whether rhythms were induced by the tissue preparation procedure could, however, not be clarified.

## Entrainment of the fetal SCN

A hallmark of circadian clocks is their ability to entrain to external *Zeitgebers*. Circadian rhythms in the embryonic SCN of different species, including humans, strongly react to different signals including light, melatonin, food, and dopamine.

Depending on the lighting conditions, rat embryos show different phasing in glucose utilization rhythms (Reppert and Schwartz, 1983). When pregnant rats are housed under normal or inverted LD cycles, the embryonic SCN entrains to the external conditions. SCNs of squirrel monkey embryos from mothers housed in different LD cycles stay in phase with the maternal rhythm (Reppert and Schwartz, 1984). However, since synaptic connections from the retina to the SCN are only formed after birth (see below) these studies suggest an indirect entrainment of embryonic SCN clocks by light, probably via signals derived from the mother and passed on through the placental barrier. One candidate for a maternal signal, which entrains the embryonic clock, is melatonin released by the pineal gland (Wurtman et al., 1964) as daily melatonin injections can reset embryonic rhythms in pregnant SCN-lesioned hamsters (Davis and Mannion, 1988). A single injection of melatonin one day before birth is sufficient to shift the phase of the offspring (Viswanathan and Davis, 1997). In pregnant pinealectomized rats arrhythmicized by constant light melatonin injections influence the phase of *Avp* and *c-fos* expression in the offspring (Houdek et al., 2014). Melatonin effects on fetal SCNs are mediated through locally expressed melatonin receptors. In capuchin monkey fetuses, melatonin 1 receptor (MT1) is strongly expressed in the SCN and suppression of maternal melatonin changes oscillating expression patterns of *Bmal1*, *Per2* and *MT1* (Torres-Farfan et al., 2006). At around midgestation, the human embryonic SCN also expresses melatonin binding sites (Reppert et al., 1988).

Periodic feeding of pregnant SCN-lesioned rats synchronizes drinking behavior in the offspring, suggesting that feeding can entrain the SCN clock of fetuses during embryogenesis (Weaver and Reppert, 1989). Even in pregnant rats housed in LD with an intact circadian clock, time-restricted feeding shifts *Per1* expression in the fetal SCN by several hours when food is restricted to a 4-h period in the inactive phase of the animals (Ohta et al., 2008). In contrast, *c-fos* and *Avp* expression patterns are not affected. However, when the circadian clocks of the rat mothers are disrupted by constant light exposure, restricted feeding resets *c-fos* and *Avp* expression of the newborns suggesting that light may be a stronger *Zeitgeber* for the fetal SCN than food-related signals (Nováková et al., 2010).

Dopamine is a potent regulator of the molecular circadian clock machinery (Yujnovsky et al., 2006). Timed treatment with SKF 38393, a dopamine receptor agonist, shifts the activity phase of the offspring from SCN-lesioned hamster mothers (Viswanathan et al., 1994; Viswanathan and Davis, 1997). Interestingly, melatonin injections have similar effects, but lead to opposite shifts in offspring activity. Dopamine and light-induced glutamatergic signaling converge on shared intracellular kinase pathways (Schurov et al., 2002; Govindarajan et al., 2006; Colwell, 2011). However, light and the melatonin phase response curves are about 12 h out of phase (Lewy et al., 1998), which may explain the opposite directional effects on offspring activity.

## Embryonic and postnatal development of SCN input pathways

After birth, light becomes the most important *Zeitgeber* to entrain circadian rhythms and also influences the maturation of the SCN itself. Irradiance information is received by melanopsin-expressing intrinsically photosensitive retinal ganglion cells (ipRGCs). ipRGCs directly sense light, but also integrate input from rod and cone photoreceptors and signal via the RHT to the SCN. Melanopsin, the photopigment of the ipRGCs, is detectable in the retina of prenatal rodents—mice E11.5 (Tarttelin et al., 2003), rats E18 (Fahrenkrug et al., 2004)—and ipRGCs become light responsive directly after gestation (Sekaran et al., 2005; Tu et al., 2005). However, at this time ipRGCs are randomly distributed throughout the ganglion cell layer and the inner nuclear layer of the mouse retina (Tu et al., 2005). Between birth and adulthood, re-organization as well as reduction of about 65% of ipRGCs occurs. During maturation ipRGCs separate into distinct regions of the inner plexiform layer. At P4, most of them are located in this region, but further separation occurs during the following days. This re-organization is accompanied by a profound loss of melanopsin-positive cells (Sekaran et al., 2005; Tu et al., 2005) that is associated with a 10-fold increase in photic sensitivity at P4-6. This increase is partially, but not solely, induced by a gradual increase in retinal melanopsin expression (Tu et al., 2005). Almost simultaneously, the first circadian rhythm of melanopsin gene (*OPN4*) expression is observed around P5 (González-Menéndez et al., 2009) indicating a further maturation of the ipRGCs.

Paralleling ipRGC development, the RHT that is principally functional directly after birth at P0 in mice (Lupi et al., 2006) and P1 in rats (Leard et al., 1994), maturates during the first 1–2 weeks after birth to reach full functionality in rats (Takahashi and Deguchi, 1983; Duncan et al., 1986). Whereas the development of the RHT of hamsters starts at P4 and reaches the adult pattern by P15, its development in rats initiates prenatally at E21–22. First connections to the ventral part of the rat SCN appear at P1, reaching maximal density at P4 and prune back to the adult pattern by P10. At that stage, the first gating of light induced *c-Fos* production is detectable in the SCN of rat pups (Bendová et al., 2004). While the maturation of the light signaling cascade is mostly finished by P10 in rats (Speh and Moore, 1993), it was shown in mice that the adult-like light response is detectable not until around P14 when the eye opening occurs (Muñoz Llamosas et al., 2000), indicating that opening the eyes and removing the light dampening cover over it enables the rodent to fully respond to the environmental light.

Whether changes in pre- or postnatal lighting conditions may interfere with the functional development of ipRGCs, the RHT or the SCN itself is not known. However, light input during postnatal phase may affect neuropeptide expression in the SCN and the circadian system. CBA/J mice, which show increased photosensitivity compared with C57Bl/6 mice, display elevated VIP and AVP levels in the SCN (Ruggiero et al., 2010) that are paradoxically associated with attenuated phase shifting behaviors (Yoshimura et al., 1994; Ruggiero et al., 2009). Additionally, constant postnatal light or darkness conditions affect the stability of circadian behavioral rhythms in mice (Canal-Corretger et al., 2000, 2001), possibly due to changes in astrocyte development in the SCN (Canal et al., 2009). Darkness exposure leads to more and larger astrocytes whereas constant light reduces SCN astrocyte numbers associated with an increased stability of circadian rhythms and overall increased running-wheel activity (Canal et al., 2009).

## Conclusion

While the structural development of the SCN is relatively well understood, the question whether endogenous circadian rhythmicity in the SCN develops before birth is still matter of debate and studies based on SCN output or clock gene expression in the SCN provide different results. Species-specific traits make it difficult to draw a generally valid conclusion and the majority of studies investigating the existence of embryonic clocks are carried out in rhythmic environments and/or with pregnant females, which have a functional circadian clock. Consequently, environmental or maternal rhythmic signals driving diurnal rhythms in the embryonic SCN cannot be excluded, and the presence of an endogenous, self-sustained fetal SCN clock cannot be demonstrated. Studying pup development in arrhythmic mothers continually housed in constant environments during pregnancy may be a feasible approach to this problem. It was shown that behavioral rhythms of pups born under such conditions are not synchronized to each other. This complicates the investigation of rhythmic gene expression in embryonic SCNs because most techniques applied to measure gene activity only allow investigating one time point per animal. An alternative approach using animals carrying circadian-controlled reporters for continuous recording of transcriptional/translational activity from one specimen was conducted. However, to clarify whether clock gene oscillations of *in vitro* SCN explants reflect rhythms that were already present *in vivo* or that were rather initiated by the tissue culture procedure, SCN tissues may be collected and cultured at different daytimes. If explant phasing was determined by *in vivo* rhythms, phasing for all groups should be roughly the same and should be independent of the preparation time. In combination with improved *in vivo* imaging techniques this may finally facilitate the determination of the emergence of SCN and peripheral tissue clock function during ontogeny and allow a clear distinction between maternal and embryo-derived rhythms.

## Conflict of interest statement

The authors declare that the research was conducted in the absence of any commercial or financial relationships that could be construed as a potential conflict of interest.
